# Relationship between the atlantodental interval and T1 slope after atlantoaxial fusion in patients with rheumatoid arthritis

**DOI:** 10.1186/s12893-020-00900-x

**Published:** 2020-11-04

**Authors:** Byeong Jin Ha, Yu Deok Won, Je Il Ryu, Myung-Hoon Han, Jin Hwan Cheong, Jae Min Kim, Hyoung-Joon Chun, Koang-Hum Bak, In-Suk Bae

**Affiliations:** 1 Department of Neurosurgery, Hanyang University Guri Hospital, 153 Gyeongchun-ro, Guri, Gyeonggi-do 11923 Republic of Korea; 2 Department of Neurosurgery, Hanyang University Medical Center, 222-1 Wangsimni-ro, Seongdong-gu, Seoul, 04763 Republic of Korea; 3 Department of Neurosurgery, Eulji University Eulji Hospital, 68, Hangeulbiseok-ro, Nowon-gu, Seoul, 01830 Republic of Korea

**Keywords:** Atlantoaxial fusion, Atlantoaxial instability, Rheumatoid arthritis, Atlantodental interval, T1 slope

## Abstract

**Background:**

Atlantoaxial fusion has been widely used for the treatment of atlantoaxial instability (AAI). However, atlantoaxial fusion sacrifices the motion of atlantoaxial articulation, and postoperative loss of cervical lordosis and aggravation of cervical kyphosis are observed. We investigated various factors under the hypothesis that the atlantodental interval (ADI) and T1 slope may be associated with sagittal alignment after atlantoaxial fusion in patients with rheumatoid arthritis (RA).

**Methods:**

We retrospectively investigated 64 patients with RA who underwent atlantoaxial fusion due to AAI. Radiological factors, including the ADI, T1 slope, Oc-C2 angle, cervical sagittal vertical axis, and C2–C7 angle, were measured before and after surgery.

**Results:**

The various factors associated with atlantoaxial fusion before and after surgery were compared according to the upper and lower preoperative ADIs. There was a significant difference in the T1 slope 1 year after surgery (p = 0.044) among the patients with lower preoperative ADI values. The multivariate logistic regression analysis showed that the preoperative ADI (> 7.92 mm) defined in the receiver-operating characteristic curve analysis was an independent predictive factor for the increase in the T1 slope 1 year after atlantoaxial fusion (odds ratio, 4.59; 95% confidence interval, 1.34–15.73; p = 0.015).

**Conclusion:**

We found an association between the preoperative ADI and difference in the T1 slope after atlantoaxial fusion in the patients with RA. A preoperative ADI (> 7.92 mm) was an independent predictor for the increase in the T1 slope after atlantoaxial fusion. Therefore, performing surgical treatment when the ADI is low would lead to better cervical sagittal alignment.

## Background

Atlantoaxial instability (AAI) in patients with rheumatoid arthritis (RA) is characterized by excessive movement between the atlas and axis. Nuchal pain is a common clinical manifestation of AAI, and severe AAI may cause radiculopathy or myelopathy due to spinal cord compression. AAI in patients with RA can have deleterious effects on both quality of life and overall health [1]. Recognizing the progressive neurological symptoms for early surgery is an important predictor of favorable patient outcomes [2–5]. The C1 lateral mass and C2 pedicle (C1LM-C2P) screw fixation technique, transarticular fixation (TAF) technique and plate and screw method of fixation of lateral masses of atlas and axis have been introduced for stabilizing AAI [6–9]. However, atlantoaxial fusion sacrifices the motion of atlantoaxial articulation.

AAI is diagnosed using lateral cervical radiography based on the presence of an anterior atlantodental interval (ADI) of ≥ 5 mm on a flexion radiograph [10, 11]. Since Coutts’ investigation in 1934, the anterior ADI has been recognized as the most sensitive gauge of atlantoaxial displacement [12]. Recently, the importance of maintaining sagittal spine balance after fusion has been emphasized. Secondary postoperative loss of cervical lordosis and even cervical kyphosis are observed in some cases, leading to nuchal pain and recurrence of instability or deterioration of neurological deficits [13–18]. The T1 slope has recently been proven as an effective index for assessing cervical spinal stability [19]. It was strongly correlated with the C2–C7 angle in the subaxial cervical spine after multi-level anterior cervical discectomy and fusion (ACDF), laminoplasty, and posterior cervical fusion [20–22]. Conversely, the postoperative C1–C2 angle has previously been reported to play an important role in postoperative alignment of the subaxial cervical spine [13]. However, only a few studies are currently investigating RA, which is one of the main causes of AAI.

To the best of our knowledge, no studies have yet investigated the association of the ADI and T1 slope with sagittal alignment after atlantoaxial fusion in patients with RA. We investigated various factors under the hypothesis that the ADI and T1 slope may be associated with sagittal alignment after atlantoaxial fusion. This study aimed to investigate and analyze the relationships among clinical factors and various radiological factors after atlantoaxial fusion in patients with AAI caused by RA.

## Methods

### Study design

We retrospectively investigated patients who underwent TAF or C1LM-C2P screw fixation for AAI in our institution from 2002 to 2014. The patients were diagnosed with RA at the Department of Rheumatology at our hospital and underwent drug therapy. All were diagnosed in accordance with the revised American College of Rheumatology 1987 Criteria [23]. Patients who had severe nuchal pain and neurological symptoms received radiological evaluation and underwent surgical treatment (TAF or C1LM-C2P screw fixation) after being diagnosed with AAI. Patients with medical records for at least 1 year after surgery were included in the analysis to obtain postoperative clinical and radiological data.

This study was approved by our Institutional Review Board (IRB; HYUH 2016-06-032-001) and conformed to the tenets of the Declaration of Helsinki. Owing to the retrospective nature of the study, the need for informed consent was waived by our IRB. All individual records were anonymized prior to analysis.

### Surgical procedures

TAF was used for AAI in our institution until 2007; thereafter, we started to use C1LM-C2P screw fixation. Thus, the patients were treated with TAF from 2002 to 2007 and with C1LM-C2P screw fixation from 2008 to 2014.

### Clinical variables

Clinical variables related to RA, including the visual analog scale (VAS) score for nuchal pain and preoperative myelopathy, were evaluated on or near the days when magnetic resonance imaging (MRI) was performed. To evaluate the effect of disease activity on surgical result after atlantoaxial fusion in the patients with RA, we included the preoperative disease activity score of 28 joints (DAS28) as a variable for the analysis which is widely used as an indicator of RA disease activity and response to treatment. Body mass index (BMI) was also analyzed.

### Radiological assessment

Cervical plain radiography was performed to obtain the flexion, neutral, and extension views before and after surgery, and radiological tests were performed for all patients at approximately 1 year after surgery. Prior to surgery, cervical computed tomography (CT) angiography was performed to confirm the course of the vertebral artery, and cervical MRI was performed for all patients to determine other ligament and spinal cord injuries. Approximately 1 year after surgery, cervical CT was performed to confirm bone union.

The ADI, T1 slope, Oc-C2 angle, C2–C7 angle, and cervical sagittal vertical axis (cSVA) were measured from cervical radiographs in the neutral position before and after surgery. One experienced neurosurgeon performed all the radiological measurements. In addition, three measurements were obtained, and the average of the three measurements was used to minimize the error. The definitions of the cervical alignment parameters used were as follows.

**Fig. 1:**
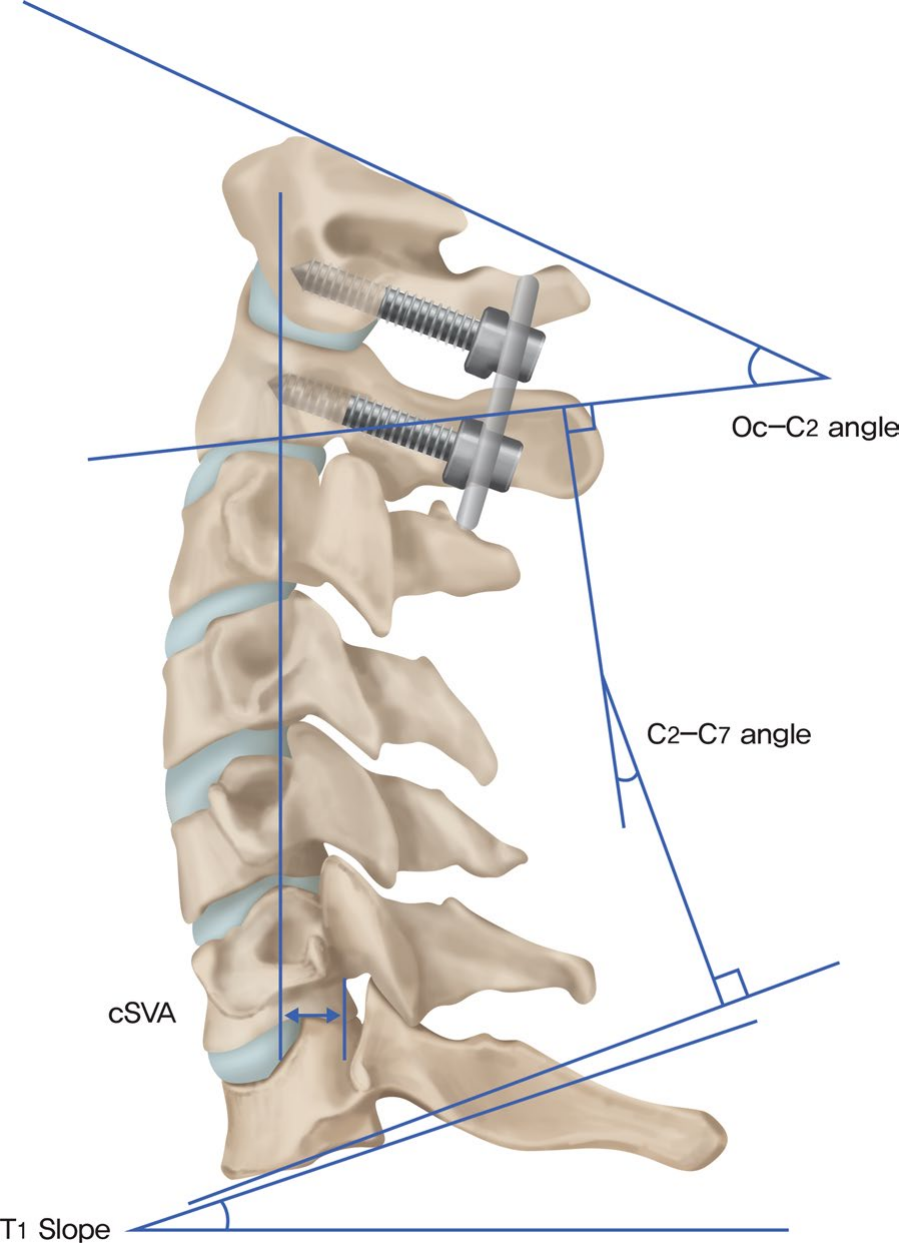
Measurement of the radiological parameters. The Oc-C2 angle, C2–C7 angle, T1 slope, and cSVA were measured between the lines shown in this figure. cSVA, cervical sagittal vertical axis

The ADI was defined as the shortest distance between the anterior margin of the dens and the posterior border of the anterior tubercle of the atlas. The T1 slope was defined as the angle between a horizontal line and the upper end plate of T1. The Oc-C2 angle was examined using the McRae line and the line tangential to the inferior aspect of the axis. The C2–C7 angle was formed by the inferior aspect of the axis and C7. The cSVA was examined on the basis of the horizontal offsets dropped by a vertical line from the mid-C2 vertebral body with respect to the mid-C7 vertebral body (Fig. 1).

### Statistical analysis

Baseline patient data were expressed as means ± standard deviations (SDs) or as medians (continuous variables) or numbers (percentages) (categorical variables). Categorical variables were examined using the χ^2^ test and continuous variables using one-way repeated-measures ANOVA. The one-way repeated-measures ANOVA was used for continuous variables to identify differences between the preoperative and postoperative (1-and 12-month) groups. The means ± SDs for the Oc-C2 angle, C2–C7 angle, T1 slope, and cSVA, classified by the median preoperative ADI, were visualized at the preoperative time points, 1 month and 12 months postoperatively.

We performed a linear regression analysis to evaluate the associations between the preoperative ADI and T1 slope difference between the preoperative and postoperative (12 months) time points. A receiver-operating characteristic curve analysis was performed to determine the optimal preoperative ADI cut-off for predicting T1 slope increase (postoperative T1 slope [12 months] > preoperative T1 slope) 1 year after atlantoaxial screw fixation for AAI.

Odds ratios (ORs) with 95% confidence intervals (CIs) were estimated using multivariate logistic regressions to determine the independent predictive factors for T1 slope increase 1 year after atlantoaxial fusion for AAI in the patients with RA.

All statistical analyses were performed using the R software version 3.3.3 (https://www.r-project.org/).

## Results

### Characteristics of the study patients

Sixty-four patients with RA from our hospital were investigated in this study over a 12-year period. The mean age at surgery was 50.9 years, and 90.6% of the patients were women. The mean preoperative ADI and T1 slope were 8.0 mm and 18.8°, respectively. Further, the median preoperative ADI was 8.15 mm. We found significant differences in the ADI and VAS score between the preoperative and postoperative (1 and 12 months) time points. Further details of the study patients are presented in Table 1.

**Table 1:** Characteristics of the study patients

Characteristics	Preoperative	Postoperative (1 month)	Postoperative (12 months)	p-value
Overall, n	64			
Female sex, n (%)	58 (90.6)			
Age, mean ± SD, year	50.9 ± 14.4			
Surgical type, n (%)				
TAF	33 (51.6)			
C1LM-C2P screw fixation	31 (48.4)			
Surgical side, n (%)				
Right	14 (21.9)			
Left	19 (29.7)			
Both	31 (48.4)			
BMI, mean ± SD, kg/m^2^	22.2 ± 2.8			
Preoperative myelopathy, n (%)	10 (15.6)			
DAS28, mean ± SD	3.6 ± 0.8			
ADI, mean ± SD, mm	8.0 ± 1.8	2.4 ± 0.7	2.3 ± 0.5	< 0.001
ADI, median (IQR), mm	8.15 (7.06–9.12)	2.42 (1.95–3.00)	2.42 (1.90–2.78)	< 0.001
Oc-C2 angle, mean ± SD	26.5 ± 9.5	29.4 ± 7.1	30.8 ± 7.1	0.010
C2-C7 angle, mean ± SD	13.3 ± 8.1	11.0 ± 5.5	12.7 ± 6.7	0.157
T1 slope, mean ± SD	18.8 ± 7.2	17.1 ± 6.4	18.3 ± 6.9	0.368
cSVA (C2-C7), mean ± SD, mm	15.5 ± 8.9	13.2 ± 7.2	12.6 ± 7.5	0.087
VAS score, median (IQR)	8 (8–9)	3 (2–3)	2 (1–2)	< 0.001

SD, standard deviation; TAF, transarticular screw fixation; C1LM-C2P, C1 lateral mass and C2 pedicle; BMI, body mass index; DAS28, disease activity score of 28 joints; ADI, atlantodental interval; IQR, interquartile range; cSVA, cervical sagittal vertical axis; VAS, visual analog scale

### Radiological parameters

The average preoperative and postoperative Oc-C2 angles in all patients were 26.5° ± 9.5° and 30.8° ± 7.1°, respectively. The mean preoperative and postoperative C2–C7 angles in all patients were 13.3° ± 8.1° and 12.7° ± 6.7°, respectively. We found significant differences in the Oc-C2 angle between the preoperative and postoperative (1 and 12 months) time points. However, there was no difference between the preoperative and postoperative C2–C7 angles, T1 slopes, and cSVA.

### Trend of the various parameters related to atlantoaxial fusion classified by the preoperative ADI

**Fig. 2:**
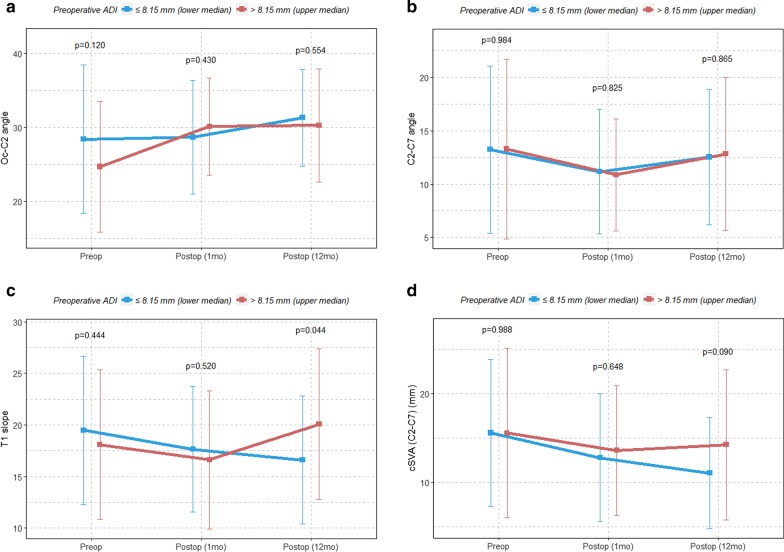
Linear graph with means ± SDs for the **a** Oc-C2 angle, **b** C2–C7 angle, **c** T1 slope, and **d** cSVA, classified by the median preoperative ADI. *SD* standard deviation, *cSVA* cervical sagittal vertical axis, *ADI* atlantodental interval

We compared the values of the various parameters related to atlantoaxial fusion between the preoperative and postoperative (1 and 12 months) time points according to the lower median preoperative ADI and upper median preoperative ADI (Fig. 2). A significant difference was noted in the T1 slope at 12 months postoperative (p = 0.044) between the lower median preoperative ADI and upper median preoperative ADI groups (Fig. 2c). The upper median preoperative ADI group had significantly higher increases in the T1 slope after atlantoaxial fusion. However, there was no significant difference in the postoperative changes in the Oc-C2 angle, C2–C7 angle, and cSVA between the upper and lower median preoperative ADI groups (Fig. 2a–d).

### Association between the preoperative ADI and T1 slope difference after atlantoaxial fusion

**Fig. 3:**
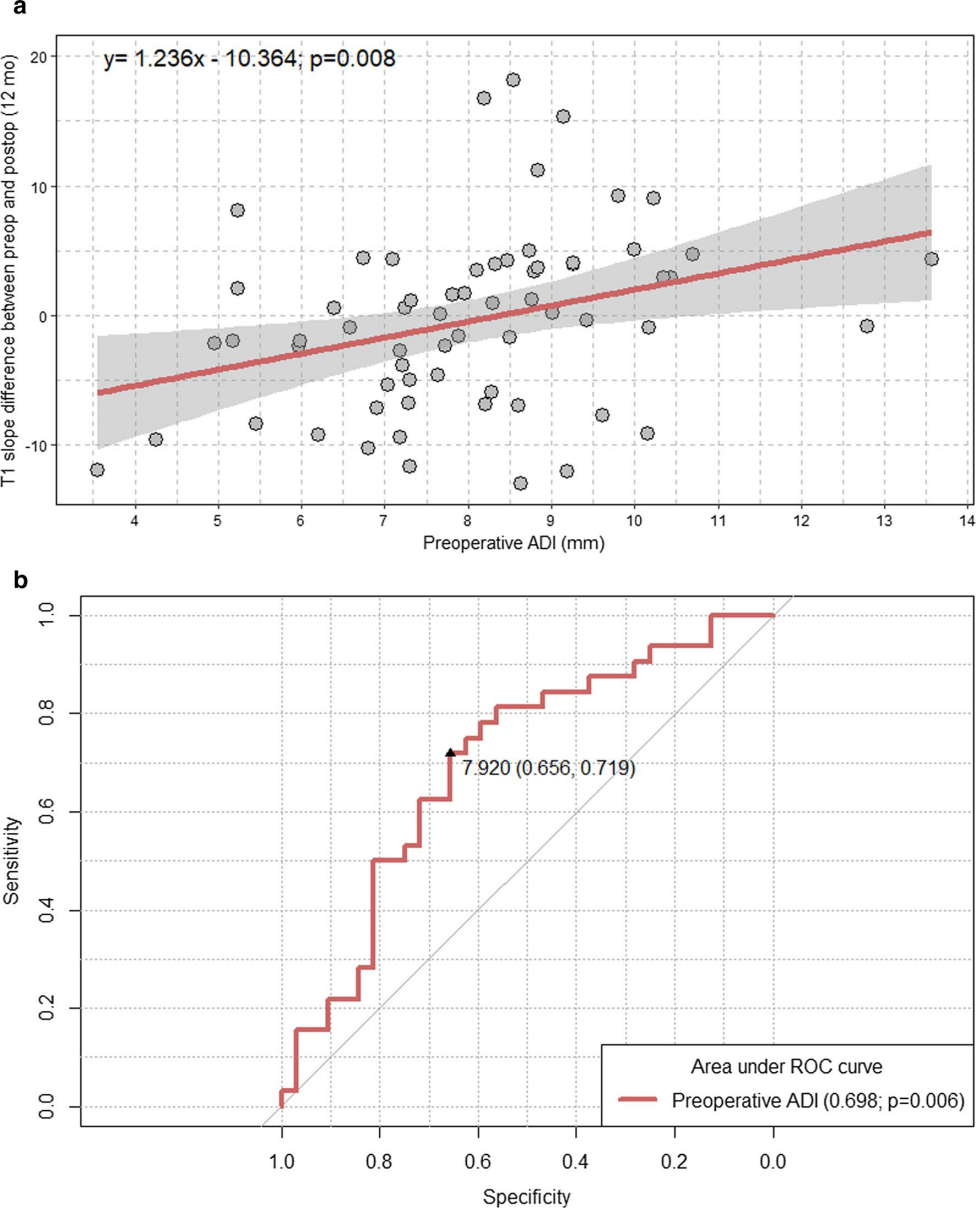
Scatter plot with a linear regression line and an ROC curve. **a** Linear regression line showing the association between the preoperative ADI and T1 slope difference between the preoperative and postoperative time points; **b** ROC curve to identify the optimal preoperative ADI cut-off for the prediction of T1 slope increase after atlantoaxial fusion. *ROC* receiver-operating characteristic, *ADI* atlantodental interval

We observed a significant positive correlation between the preoperative ADI and T1 slope difference after atlantoaxial fusion (Fig. 3a). The linear regression analysis demonstrated an approximate of 1.2-degree increase in the T1 slope difference per 1-mm preoperative ADI increase (β = 1.236; p = 0.008). The optimal cut-off value of the preoperative ADI for prediction of T1 slope increase 1 year after atlantoaxial fusion was 7.920 mm (AUC = 0.698; sensitivity = 71.9%; specificity = 65.6%; p = 0.006) (Fig. 3b).

### Independent predictive factor for T1 slope increase after atlantoaxial fusion

We employed a multivariate logistic regression model to determine the independent predictive factors for T1 slope increase 1 year after atlantoaxial fusion for AAI in the patients with RA. The multivariate logistic regression analysis identified a higher preoperative ADI (> 7.92 mm) as an independent predictor of T1 slope increase 1 year after atlantoaxial fusion (OR, 4.59; 95% CI, 1.34–15.73; p = 0.015) (Table 2). We found a nearly 4.6-fold higher T1 slope increase in the higher preoperative ADI group (> 7.92 mm) than in the lower preoperative ADI group.

**Table 2:** Multivariate logistic regression analysis of TI slope increase 1 year after atlantoaxial fusion for atlantoaxial instability in the patients with rheumatoid arthritis

	Multivariate logistic regression analysis		
Variable	OR	95% CI	p-value
Sex			
Female		Reference	
Male	14.21	0.44–458.84	0.134
Age	0.97	0.93–1.02	0.215
Preoperative myelopathy	1.87	0.33–10.51	0.477
Preoperative VAS score	1.17	0.59–2.34	0.652
DSA28	0.92	0.41–2.08	0.841
Preoperative ADI			
≤ 7.92 mm		Reference	
> 7.92 mm	4.59	1.34–15.73	0.015
Preoperative Oc-C2 angle	1.00	0.94–1.06	0.985
Preoperative C2–C7 angle	0.93	0.84–1.02	0.107
Preoperative cSVA (C2–C7)	1.01	0.94–1.09	0.726

*OR* odds ratio, *CI* confidence interval, *VAS* visual analog scale, *DAS28* disease activity score of 28 joints, *ADI* atlantodental interval, *Oc-C2* occipito-C2, *cSVA* cervical sagittal vertical axis

## Discussion

In the present study, we retrospectively investigated various factors under the hypothesis that the ADI and T1 slope may be associated with sagittal alignment after atlantoaxial fusion. We found an association between the preoperative ADI and difference in the T1 slope after atlantoaxial fusion in the patients with RA. A preoperative ADI higher than 7.92 mm was an independent predictor for T1 slope increase after atlantoaxial fusion. We found a nearly 4.6-fold higher T1 slope increase in the higher preoperative ADI group (> 7.92 mm) than in the lower preoperative ADI group.

Previous studies on subaxial cervical spinal changes after atlantoaxial fusion have reported limited findings with regard to predicting these changes in patients with RA because they enrolled patients with various etiologies of C1–C2 instability. In the present study, we evaluated patients with AAI due to RA who underwent TAF or C1LM-C2P screw fixation. Therefore, our study represents a more homogeneous group than do previous studies. Changes in the subaxial cervical spine can develop not only as a natural course of RA but also as a consequence of upper cervical fusion or disruption of the extensor muscles involved in posterior cervical surgery [24–26]. For these reasons, we focused on atlantoaxial fusion for AAI in patients with RA. To our knowledge, this study is the first to demonstrate the relationship between the preoperative ADI and postoperative changes in the T1 slope after atlantoaxial fusion in patients with RA.

C1-C2 arthrodesis has been widely used in the treatment of AAI in patients with long-standing RA. The C1LM-C2P screw fixation technique and TAF technique have been introduced to stabilize AAI [6, 7]. However, atlantoaxial fusion limits the motion of the atlantoaxial spine. In addition, we often encounter postoperative subaxial alignment changes in some cases, which can be a cause of neck pain or neurologic impairment [13–18]. Kyphotic changes in the subaxial cervical spine are one of the adverse events following atlantoaxial fusion. Approximately 33%-48% of all patients who undergo atlantoaxial fusion develop postoperative kyphosis or swan-neck deformity of the lower cervical spine. Yoshimoto et al. reported that 42% of their patients who underwent atlantoaxial fusion showed progression of kyphosis in the subaxial cervical spine, which is attributable to atlantoaxial fusion in a hyperextended position [18, 27–29]. However, these results are limited in significance because these previous studies included a variety of surgeries and diseases.

The C1-C2 fixation angle has been emphasized as a key factor to regulate cervical subaxial alignment in atlantoaxial fusion in previous reports [14, 17, 18, 30, 31]. In asymptomatic individuals, there was a negative linear correlation between the angles of C1–C2 and C2–C7 [30, 31]. For patients with AAI, C1–C2 arthrodesis in a hyperlordotic position could cause sagittal kyphosis of the lower cervical spine. The subaxial kyphosis is more frequently developed as the C1–C2 fixation angle increases from surgery. Therefore, atlantoaxial fixation in excessive lordotic alignment in a hyperextended position should be avoided to prevent subaxial malalignment postoperatively [14, 18].

Previous studies have reported correlations among several cervical alignment parameters, including the T1 slope, C2–C7 angle, and Oc-C2 angle [32]. Various types of cervical surgery were associated with changes in postoperative sagittal balance and postoperative symptoms. Kwon et al. reported that the C2–C7 SVA after two-level ACDF was affected more significantly by the SVA and C2–C7 angle than by the T1 slope [22]. Knott et al. suggested that the T1 slope is the most important predictor of the C2–C7 SVA and recommended to perform cervical radiography at an upright position when the T1 slope is below 13° or above 25° [19]. Kim et al. measured cervical sagittal alignment after laminoplasty and reported that cervical kyphotic deformity at the 2-year postoperative follow-up increased with increasing preoperative T1 slope [21]. Hyun et al. suggested that the T1 slope and C2–C7 lordosis mismatch is a cervical analog for cervical lordosis and thoracic lumbar pelvic incidence [33]. These studies revealed that a deformity of the upper cervical spine is compensated by the subaxial cervical spine, including the T1 slope.

In addition, several studies have reported associations between clinical outcomes and alignment of the cervical spine after cervical spinal surgery. Naderi et al. suggested that an abnormal cervical curvature was associated with less improvement in neurological symptoms after surgery [34]. In a double-blinded randomized trial, improvement in cervical sagittal alignment was not correlated with clinical outcomes after anterior cervical fusion; however, an improved segmental angle was associated with an improvement in clinical outcomes [35]. Guérin et al. also reported that improvement in the segmental angles, as opposed to that in the cervical lordotic angles, is correlated with improvement in clinical symptoms after cervical disc replacement [36]. Improvement in cervical sagittal alignments was associated with better clinical outcomes after cervical spinal surgery.

In our study, we demonstrated a significant positive correlation between the preoperative ADI and T1 slope difference after atlantoaxial fusion. A higher preoperative ADI (> 7.92 mm) was related to more increases in the T1 slope after surgery. The T1 slope is well known parameter that may be easily used in evaluating sagittal balance in particular situations full column radiographs are not available [19]. It is constant morphological values within an individual and positively correlated with subaxial lordosis to maintain sagittal balance of the cervical spine [20, 21]. Moreover, an increasing T1 slope has been shown to correlate with greater sagittal malalignment of the dens significantly [19, 32].

The T1 slope significantly increased more in the patients with a higher preoperative ADI, which adversely affected the sagittal alignment of the cervical spine after atlantoaxial fusion. Moreover, the increasing T1 slope significantly correlated with sagittal malalignment of the cervical spine. For these reasons, surgeons should consider cervical sagittal balance when fixating C1–C2 screws during surgery in patients with higher preoperative ADIs.

Our study aimed to investigate various factors under the hypothesis that the ADI and T1 slope may be associated with sagittal alignment after atlantoaxial fusion for AAI among patients with RA. When the preoperative ADI was ≥ 7.92 mm, the patients who underwent surgical treatment showed significantly more increases in the T1 slope. This suggests that AAI due to RA causes degenerative changes in the subaxial cervical spine as well as atlantoaxial lesions, which might affect cervical sagittal alignment. Therefore, performing surgical treatment in patients with lower ADIs would lead to better sagittal alignment and less pain. The ADI in patients with AAI will increase over time. Surgeons need to monitor ADI changes closely and administer appropriate treatments in response to such changes. Nuchal pain caused by cervical spinal instability can be resolved after cervical fusion. However, further improvement in symptoms can be expected when sagittal alignment is carefully considered. For this reason, the appropriate cervical angle should be considered after fixating the head in the prone position before surgery; the appropriate angle should also be considered for rod fixation after screw fixation of the C1 and C2 during surgery.

This study has several limitations. First, it was a retrospective study of a relatively small sample of patients. Second, the retrospective nature of the analysis in this study might have introduced some patient selection bias. Third, the only symptom that was investigated before and after surgery was neck pain, as measured by the VAS score. Thus, the relationship between the T1 slope and clinical outcomes might need further assessment. We also did not evaluate the complications after surgery. These limitations indicate the need for further research and clinical studies in this field. Prospective studies on a larger sample are needed. Subsequent prospective studies should not only investigate the cervical sagittal angle but also analyze the overall spinal angle.

## Conclusion

This study confirmed that the preoperative ADI and changes in the T1 slope are correlated with each other after atlantoaxial fusion for AAI in patients with RA. Surgeons need to consider cervical sagittal alignment more in patients with higher preoperative ADIs when performing atlantoaxial fusion. Further, when patients with RA develop AAI, changes in the ADI should be closely observed using radiography. If there are neurological symptoms in patients with RA and AAI, surgical treatment should be considered before the ADI increases too much. Achieving appropriate sagittal changes after surgery would be effective for achieving cervical sagittal alignment and lead to better clinical outcomes.

## Data Availability

The datasets used and analyzed during the current study are available from the corresponding author on reasonable request.

## Abbreviations

AAI: Atlantoaxial instability
RA: Rheumatoid arthritis
ADI: Atlantodental interval
C1LM-C2P: C1 lateral mass and C2 pedicle
TAF: Transarticular fixation (TAF)
ACDF: Anterior cervical discectomy and fusion
VAS: Visual analogue scale
MRI: Magnetic resonance imaging
BMI: Body mass index
CT: Computed tomography
cSVA: Cervical sagittal vertical axis
SD: Standard deviation
OR: Odds ratios
CI: Confidence intervals
